# Polyoxometalate K_6_[P_2_Mo_18_O_62_] Inactivates *Escherichia coli* O157:H7 by Inducing recA Expression and Apoptosis-like Bacterial Death

**DOI:** 10.3390/ijms241411469

**Published:** 2023-07-14

**Authors:** Shaoling Lin, Zhongjing Lin, Feng Zhou, Dehua Wang, Baodong Zheng, Jiamiao Hu

**Affiliations:** 1 College of Food Science, Fujian Agriculture and Forestry University, Fuzhou 350002, China; shaoling.lin@fafu.edu.cn (S.L.); zhongjing.lin@fafu.edu.cn (Z.L.);; 2 College of Life Sciences, University of Leicester, Leicester LE1 7RH, UK

**Keywords:** polyoxometalates, K_6_[P_2_Mo_18_O_62_], bacterial apoptosis-like death, recA

## Abstract

Polyoxometalates have emerged as promising bactericidal agents. In the current study, the bactericidal activity of polyoxometalate K_6_[P_2_Mo_18_O_62_] against *Escherichia coli* (*E. coli*) O157:H7 and its possible underlying mechanisms were explored. The obtained results demonstrated that K_6_[P_2_Mo_18_O_62_] could effectively kill *E. coli* O157:H7 at millimolar levels. Moreover, K_6_[P_2_Mo_18_O_62_] treatment also induced significant increases in recA protein expression and further triggered characteristic apoptosis-like bacterial death events such as DNA fragmentation and phosphatidylserine exposure. In conclusion, polyoxometalate K_6_[P_2_Mo_18_O_62_] possesses a desirable antibacterial activity, and induction of bacterial apoptosis-like death might be involved in its underlying bactericidal mechanisms.

## 1. Introduction

Antimicrobial resistance presents a serious concern worldwide. In recent decades, a variety of novel strategies have been adopted to diversify antibacterial agents. For instance, a great number of natural bioactive compounds have been reported to possess antibacterial activity [1]. Meanwhile, a considerable amount of effort has also been made to identify synthetic compounds with a significant and/or broad spectrum bactericidal activity, such as nanoparticles and organic–inorganic hybrid compounds [2,3,4,5].

In particular, polyoxometalates, a group of discrete polynuclear early transition metal oxide clusters [6], have been widely reported as potential antibacterial agents against numerous food-borne bacteria [7]. Moreover, studies also revealed that polyoxometalates not only exert a significant bactericidal activity themselves, but also exhibit strong synergistic antibacterial effects together with conventional antibiotics [8,9].

Currently, polyoxometalate–protein and polyoxometalate–enzyme interactions are believed to play crucial roles in mediating the bactericidal activity [10]. In some cases, polyoxometalates can even form covalent interactions with biomacromolecules to affect their normal functions. Thus, polyoxometalates can interfere with a diverse range of functions of proteins and enzymes which are indispensable for bacterial survival and growth [11]. Notably, recent studies also revealed that a number of polyoxometalates could exert phosphatase activities [12,13,14,15,16], showing the diversity of the interactions between biomolecules and polyoxometalates.

Bacterial apoptosis-like death, also known as bacterial programmed cell death, is a kind of bacterial response to environmental stress, which is mainly mediated by the recA-lexA pathway and the mazEF-mediated pathway in *Escherichia coli* (*E. coli*) [17]. Studies have further revealed that these two mechanisms could work together in bacteria to determine their fate. For instance, the mazEF-mediated pathway reduces *recA* mRNA levels. Notably, DNA damage has been identified as the main cause of recA-mediated bacterial apoptosis-like death [18]. It has been reported that the recA expression can increase 17-fold within 10 min of UV irradiation [19]. Indeed, a number of bactericidal compounds (e.g., antibiotics) were found to induce the expression of recA as well as consequent physiological and biochemical hallmarks of bacterial apoptosis-like death [20,21,22,23].

However, to the best of our knowledge, it is still unknown whether bacterial apoptosis-like death is associated with the antibacterial effects of polyoxometalates. Therefore, in the current study, the polyoxometalate K_6_[P_2_Mo_18_O_62_] was synthesized and its bactericidal activity against *E. coli* O157:H7 (one the most common bacterial strains to cause food-borne illnesses in people [24]) was determined. Moreover, the possible involvement of bacterial apoptosis-like death in its underlying bactericidal mechanisms was also investigated.

## 2. Results

### 2.1. Synthesis and Characterization of K_6_[P_2_Mo_18_O_62_]

**Figure 1:**
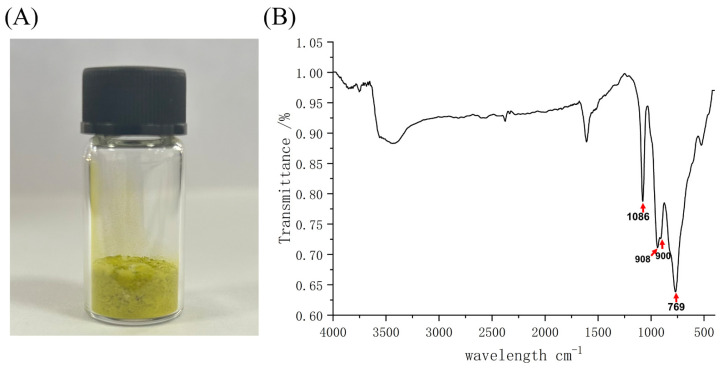
Synthesis and characterization of polyoxometalate K_6_[P_2_Mo_18_O_62_]. (**A**) Picture of as-synthesized K_6_[P_2_Mo_18_O_62_] powder. (**B**) FTIR spectrum of as-synthesized K_6_[P_2_Mo_18_O_62_].

The polyoxometalate K_6_[P_2_Mo_18_O_62_] was synthesized according to the literature [25]. As expected, the resultant yellow powder was obtained as shown in Figure 1A. Furthermore, the infrared spectra of the as-synthesized powders showed the characteristic peaks of polyoxometalate K_6_[P_2_Mo_18_O_62_] located at 769, 900, 908, and 1086 cm^−1^, which are the tensile vibration characteristic peaks of Mo-O_c_-Mo, Mo-O_b_-Mo, Mo-O_d_, and P-O_a_ (O_a_ represents those coordinated to P and to Mo atoms, O_b_ represents those coordinated to Mo atoms whose MoO_6_ octahedron are corner-shared, O_c_ represents those coordinated to Mo atoms whose octahedron are side-shared, and O_d_ represents the terminal oxygen atoms), respectively (Figure 1B). Taken together, the obtained results suggest that the polyoxometalate K_6_[P_2_Mo_18_O_62_] was synthesized successfully.

### 2.2. Antibacterial Effects of K_6_[P_2_Mo_18_O_62_] against E. coli O157:H7

**Figure 2:**
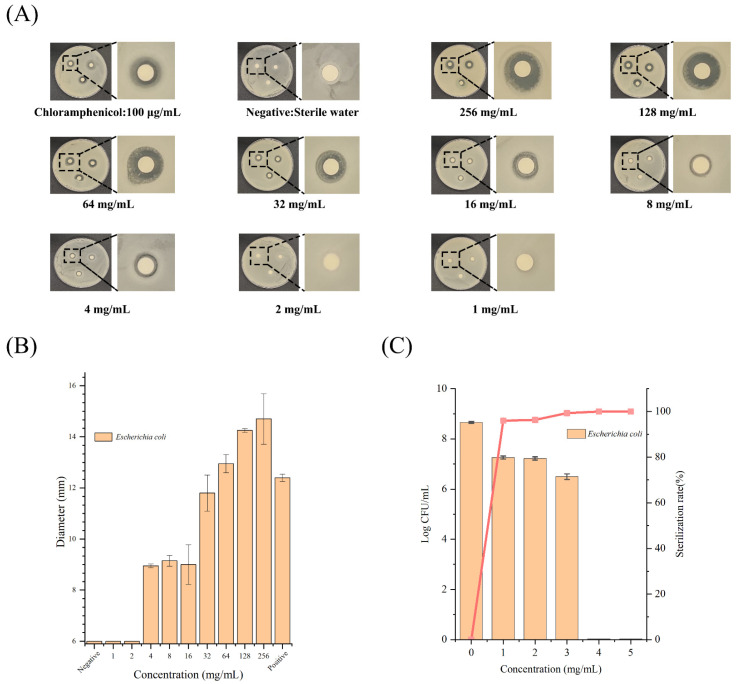
Bactericidal effects of polyoxometalate K_6_[P_2_Mo_18_O_62_] against *E. coli*. (**A**) The killing effect of polyoxometalate K_6_[P_2_Mo_18_O_62_] on *E. coli* O157:H7 determined by a Kirby–Bauer disk diffusion test. The 6 mm filter paper disks impregnated with sterile water (negative control), chloramphenicol (positive control) and K_6_[P_2_Mo_18_O_62_] at different concentrations. (**B**) The inhibition zone diameter size. (**C**) The killing effect of polyoxometalate K_6_[P_2_Mo_18_O_62_] on *E. coli* O157:H7 determined by the agar contact method. The colony numbers of *E. coli* O157:H7 were counted on plates containing K_6_[P_2_Mo_18_O_62_] at different concentrations.

As shown in Figure 2A, the results from the Kirby–Bauer disk diffusion test showed a clear circular area around the discs impregnated with K_6_[P_2_Mo_18_O_62_] at concentrations of 4 mg/mL or above. The inhibition zone diameters further increased with the increase in K_6_[P_2_Mo_18_O_62_] concentration (Figure 2B). Similarly, the solid contact test also supported the above findings. As can been seen from Figure 2C, the inhibitory effect against *E. coli* could reach 95.99 ± 0.86% on the LB agar plate containing K_6_[P_2_Mo_18_O_62_] at 1 mg/mL. Further increases in the concentration of K_6_[P_2_Mo_18_O_62_] resulted in better inhibitory effects on *E. coli*. (the CFU of bacteria decreased by 2.03 and 2.16 logarithms, respectively, when the K_6_[P_2_Mo_18_O_62_] concentration was 2 and 3 mg/mL). When the concentration reached 4 mg/mL in the agar plate, nearly 100% of bacteria could not survive, evidenced by the fact that no bacterial colony was observed on the plate after incubation at 37 °C for 12 h. The results showed that K_6_[P_2_Mo_18_O_62_] demonstrated a desirable killing activity against the typical food-borne pathogen *E. coli.* O157:H7.

### 2.3. K_6_[P_2_Mo_18_O_62_] Possesses Phosphatase Activity

**Figure 3:**
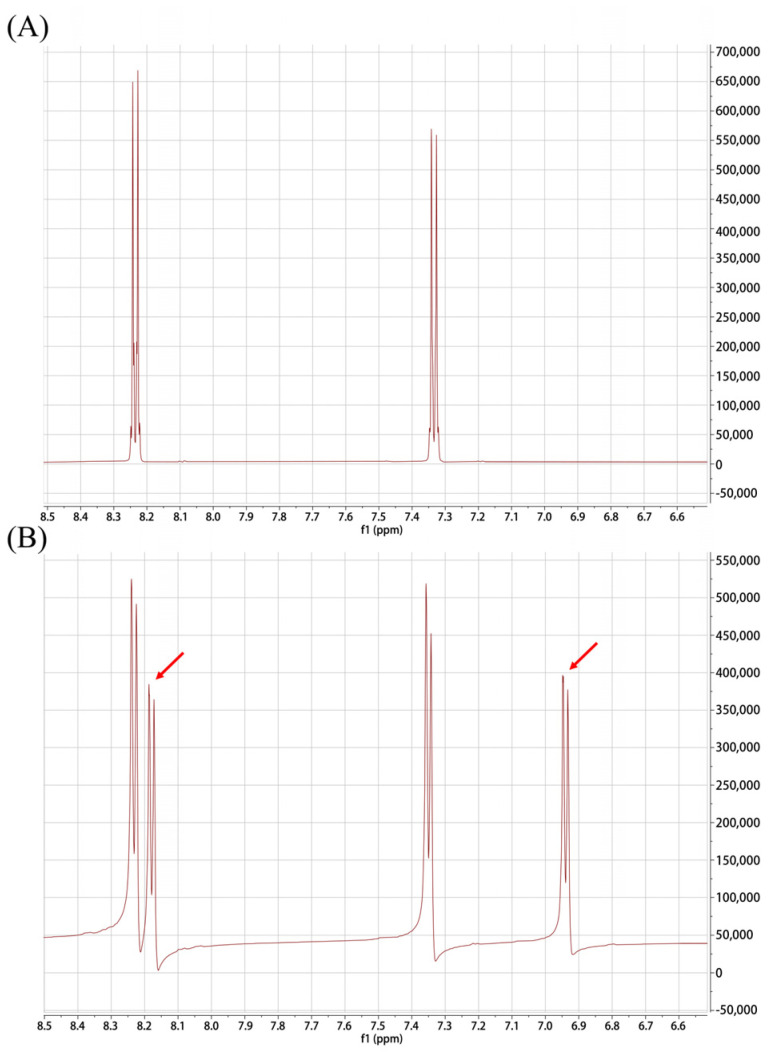
^1^H NMR spectra of 4-nitrophenyl phosphate (NPP) incubated without (**A**) or with (**B**) polyoxometalate K_6_[P_2_Mo_18_O_62_]. The red arrow indicates the new resonances caused by the formation of p-nitrophenol (NP).

As reported in a previous study [12], the potential phosphatase activity of K_6_[P_2_Mo_18_O_62_] was evaluated by determining the cleavage of the phosphate bond of the DNA analogue 4-nitrophenyl phosphate (NPP). As shown in Figure 3, the ^1^H NMR spectra showed that NPP incubation with K_6_[P_2_Mo_18_O_62_] resulted in new resonances (6.89 ppm and 8.18 ppm) in addition to the resonances of the orthoprotons of NPP (7.34 ppm and 8.22 ppm), showing the formation of a hydrolysis product (p-nitrophenol (NP)) [14]. Thus, the experimental results indicated that the phosphate ester bonds in the DNA analogue NPP could be cleaved by K_6_[P_2_Mo_18_O_62_], highlighting its capability to induce DNA damage.

### 2.4. K_6_[P_2_Mo_18_O_62_] Treatment Induced a Significant Increase in the recA Expression in E. coli O157:H7

**Figure 4:**
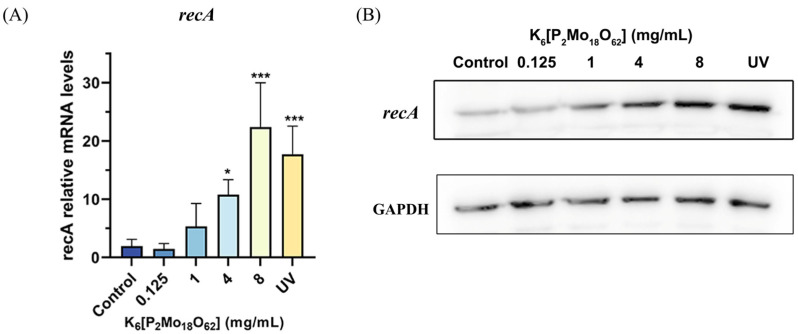
Polyoxometalate K_6_[P_2_Mo_18_O_62_] treatment induced recA expression in *E. coli*. (**A**) The effect of K_6_[P_2_Mo_18_O_62_] treatment on the mRNA level of *recA* in *E. coli*. (**B**) The effect of K_6_[P_2_Mo_18_O_62_] treatment on the expression of recA protein in *E. coli*. * *p* < 0.05, *** *p* < 0.001 compared to control group.

Next, the induction effects of K_6_[P_2_Mo_18_O_62_] on the recA expression in *E. coli* was explored using real-time PCR. As can be seen from Figure 4A, the *recA* transcription was significantly elevated upon K_6_[P_2_Mo_18_O_62_] treatment.

Western blotting was also applied to further verify the changes in the recA protein expression in *E. coli* upon K_6_[P_2_Mo_18_O_62_] treatment. As shown in Figure 4B, K_6_[P_2_Mo_18_O_62_] treatment resulted in obvious increases in recA protein compared with the non-treatment control group, while K_6_[P_2_Mo_18_O_62_] at 4 mg/mL or above demonstrated a similar induction effect to UV irradiation, which is a well-known recA inducer in *E. coli*. In summary, the results from both transcriptional and protein levels showed K_6_[P_2_Mo_18_O_62_] could alter the recA expression.

### 2.5. K_6_[P_2_Mo_18_O_62_] Treatment Induced Apoptosis-Like Bacterial Death Events in E. coli O157:H7

**Figure 5:**
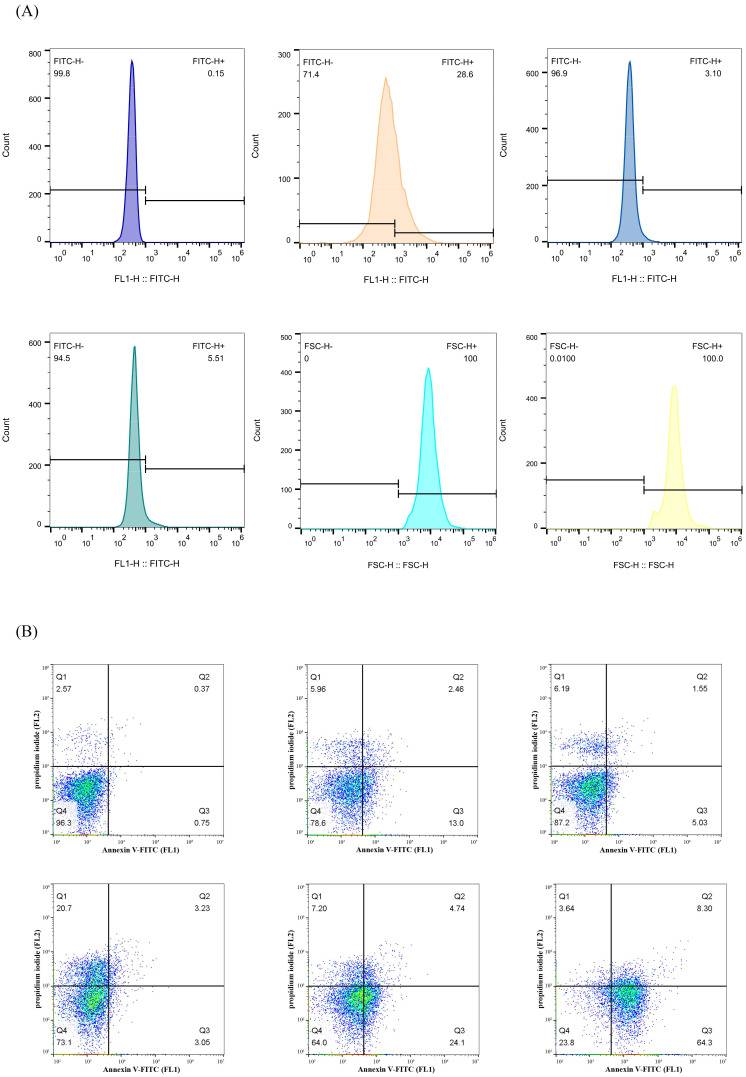
Polyoxometalate K_6_[P_2_Mo_18_O_62_] treatment induced characteristic apoptosis-like bacterial death events. (**A**) Quantitation of TUNEL staining by flow cytometry. (**B**) Quantitation of annexin V-FITC/propyl iodide staining by flow cytometry.

The protein recA has been documented as an important regulator of the apoptotic demise of bacteria, which is characterized by DNA fragmentation and membrane depolarization [17]. As shown in Figure 5A, the flow cytometry analysis of bacteria with TUNEL staining showed that treatment with K_6_[P_2_Mo_18_O_62_] yielded obvious increases in the percentages of TUNEL positive cells, indicating that fragmentation of *E. coli* DNA occurred upon treatment with the death-inducing polyoxometalate. Next, we also performed a flow cytometry analysis with Annexin V-FITC staining to detect phosphatidylserine exposure, a characteristic event of membrane depolarization. The obtained results also showed bacterial population in the lower right (Q3) quadrant, which indicated that FITC-annexin V-positive/PI-negative cells dramatically increased following K_6_[P_2_Mo_18_O_62_] treatment, suggesting phosphatidylserine externalization at the outer layer of the cytoplasmic membrane in *E. coli* cells (Figure 5B). Taken together, the results revealed that, upon K_6_[P_2_Mo_18_O_62_] treatment, *E. coli* showed the characteristics of apoptosis-like bacterial death events.

## 3. Discussion

Polyoxometalates represent a large class of anionic clusters, consisting of transitional metal oxides with a wide variety of physical and chemical properties. In recent decades, the interest in polyoxometalate-related studies has steadily expanded due to the variety of their functionalities. Particularly, a number of studies have focused on the potential of polyoxometalates in the battle against bacteria. For instance, Nadiia Gumerova et al. reported the antibacterial activity of 29 different polyoxometalates and found that a Preyssler-type polyoxometalate ([NaP_5_W_30_O_110_]^14−^) and Dawson-type polyoxometalates ([P_2_W_18_O_62_]^6−^, [(P_2_O_7_)Mo_18_O_54_]^4−^, [As_2_Mo_18_O_62_]^6−^ and [H_3_P_2_W_15_V_3_O_62_]^6−^) showed promising antibacterial activity against *M. catarrhalis* [26]. Indeed, more studies also suggested that polyoxometalates could synergistically kill the bacteria with traditional antibiotics. For example, Inoue et al. reported that polyoxometalates (K_6_[P_2_W_18_O_62_]·14H_2_O, K_4_[SiMo_12_O_40_]·3H_2_O, and K_7_[PTi_2_W_10_O_40_]·6H_2_O) showed strong sensitizing effects against methicillin-resistant *Staphylococcus aureus* (MRSA) and vancomycin-resistant *Staphylococcus aureus* (VRSA) strains [27]. These findings highlighted the promising potential of polyoxometalates as anti-bacterial agents. Here, a Dawson-type polyoxometalate K_6_[P_2_Mo_18_O_62_] with a desirable bactericidal activity against *E. coli* O157:H7 was prepared by hydrothermal synthesis and characterized by infrared spectroscopy. The obtained infrared spectrum was consistent with the properties of H_6_[P_2_Mo_18_O_62_] reported by Ding et al. [28], indicating K_6_[P_2_Mo_18_O_62_] was successfully synthesized.

Although it is widely accepted that the mechanisms underlying the bactericidal activity of polyoxometalates could be attributed to their interaction with proteins and enzymes [10], it is worth mentioning that phosphatase activity has been identified in a range of polyoxometalates [12,13,14,15,16] in the last decade. Thus, their potential DNA-damaging effects should not be overlooked, which have been identified as the main cause of recA-mediated bacterial apoptosis-like death [18].

Apoptosis is a programmed cell death which was believed to occur only in eukaryotes. However, in recent decades, increasing evidence has demonstrated that events similar to the characteristic hallmarks of apoptosis also take place in bacteria, suggesting that bacteria also contain basic cell death programs, known as apoptosis-like death. Indeed, growing evidence shows a range of death-inducing stress (e.g., antibacterial agent treatment, UV irradiation, etc.) could trigger apoptosis-like processes in bacteria. For example, it has been revealed that a complex of alpha-lactalbumin (ALA) and oleic acid isolated from human milk with antimicrobial activity could induce *S. pneumoniae* to display phenotypic traits of apoptosis. Here, the polyoxometalate [P_2_Mo_18_O_62_] was also found to induce the over-expression of recA (a key protein controlling bacterial apoptosis-like death) and further resulted a range of apoptosis-like bacterial death events such as DNA fragmentation and phosphatidylserine exposure. Therefore, induction of bacterial apoptosis-like death might be involved in its underlying bactericidal mechanisms.

Admittedly, here, the association between polyoxometalates and bacterial apoptosis-like death was only evaluated in *E. coli* O157:H7 upon K_6_[P_2_Mo_18_O_62_] treatment. Indeed, a comprehensive assessment of the pro-apoptotic properties of polyoxometalates with different structures in various food-borne bacteria strains could provide more insight, especially considering a range of polyoxometalates have been reported with better properties (e.g., a higher killing activity against bacteria (lower MIC at micromolar level) [26], a lower toxicity against mammalian cells (higher IC_50_) [29], etc.) than K_6_[P_2_Mo_18_O_62_]. In addition, the latest evidence also suggests that bacterial apoptosis-like death plays a crucial role in biofilm development [30]. Overall, investigating the roles of bacterial programmed cell death in the bactericidal action of polyoxometalates could provide useful information for exploring novel anti-bacterial agents in the future.

## 4. Materials and Methods

### 4.1. Chemicals, Reagents and Bacterial Strains

Sodium molybdate dihydrate (Na_2_MoO_4_·2H_2_O, 99%), phosphoric acid (H_3_PO_4_, 85%), hydrochloric acid (HCl, SCR, 36%~38%), potassium bromide (KBr, 90%), and 3-(Trimethylsilyl)propionic-2,2,3,3-d_4_ acid sodium salt ((CH_3_)_3_SiCD_2_CD_2_CO_2_Na, 98%) were purchased from Macklin. *Escherichia coli* (*E. coli*) O157:H7 was purchased from China from the Industrial Culture Collection.

### 4.2. Synthesis and Characterization of K_6_[P_2_Mo_18_O_62_]

The synthesis of polyoxometalate K_6_[P_2_Mo_18_O_62_] was performed according to our previous publication [25]. For each batch of synthesis, 40 g of sodium molybdate dihydrate, 6 mL of phosphoric acid, 33 mL of hydrochloric acid, and 40 g of potassium bromide were used. IR spectroscopy was applied to confirm the successful synthesis of K_6_[P_2_Mo_18_O_62_].

### 4.3. Antibacterial Activity Tests

#### 4.3.1. Kirby–Bauer Disk Diffusion Test

The killing effect of polyoxometalate K_6_[P_2_Mo_18_O_62_] on *E. coli* O157:H7 was firstly evaluated using the Kirby–Bauer disk diffusion method according to the method in the literature [31]. In brief, *E. coli* in the logarithmic growth phase was evenly spread over LB agar plates, and sensitivity test paper discs impregnated with different concentrations of K_6_[P_2_Mo_18_O_62_] were pasted on the surface of the agar plates. Each test was repeated three times with sterile water as a negative control and chloramphenicol as a positive control. After incubation at 37 °C for 24 h, the inhibition diameter was recorded by a Vernier caliper and an image was captured with a digital camera.

#### 4.3.2. Agar Contact Method

The killing effect of polyoxometalate K_6_[P_2_Mo_18_O_62_] on *E. coli* O157:H7 was also evaluated using the agar contact method according to the literature with some modifications [32].

In brief, the LB agar plates (molten agar medium) were prepared with incorporation of varying concentrations of the antimicrobial agents (chloramphenicol or K_6_[P_2_Mo_18_O_62_]). Then, the bacteria were evenly spread on LB plates. After incubation at 37 °C for 24 h, the inhibition rate was calculated by counting the decrease in the colony number when compared to the negative control plate (without antimicrobial agents). Each test was repeated three times.

### 4.4. Real-Time PCR

*E. coli* was cultured in liquid LB broth and then treated with K_6_[P_2_Mo_18_O_62_] for 3 h at 37 °C. The bacterial total RNA was isolated using an E.Z.N.A.^®^ Bacterial RNA Kit (Omega Bio-tek). The RNA concentration was determined using a NanoDrop™ 2000 Spectrophotometer, before isolated RNA (1 μg) was reverse-transcribed to cDNA using a PrimeScript™ RT Reagent Kit (Takara, Shiga, Japan).

The resultant cDNA was then used as a template for real-time PCR, which was performed on a qTOWER³ (Analytik Jena AG, Jena, Germany) under the following settings: an initial cycle at 95 °C for 2 min, followed by 40 cycles of 95 °C for 15 s and 60 °C for 50 s with the following melt curve determination using 2× SYBR green PCR Master Mix. The primer sets used in the current study were: recA-F: GCGAAATCGGCGACTCTC; recA-R: ATCAGCAGC GTGTTGGAC; 16S-F: GCAAGCGGACCTCATAAA; and 16S-R: ATTCACCGTGGCATTCTG.

### 4.5. Western Blot

The Western blot assay was carried out according to the method of Grinholc et al. [33]. In short, *E. coli* was treated according to the description above, and then the protein was extracted using a Bacterial Protein Extraction Kit (Sangon Biotech, Shanghai, China). The protein was quantified and separated by SDS-PAGE electrophoresis before the gel was transferred to a PVDF membrane. The PVDF membrane was immediately blocked with 5% skimmed milk at room temperature for 1 h and incubated with *E. coli recA* polyclonal antibody (Abnova, 1:3000) at 4 °C for 12 h. Subsequently, the PVDF membrane was incubated with Goat anti-rabbit IgG H&L/HRP antibody (Bioss, 1:3000) at room temperature for 1 h. Then, an enhanced ECL chemiluminescence substrate (Biosharp, Hefei, China) was used to visualize the protein band. GAPDH protein was used as an internal reference protein.

### 4.6. Flow Cytometric Analysis

#### 4.6.1. Flow Cytometric Analysis of the TUNEL Assay

*E. coli* was treated according to the description above, and then collected by centrifugation at 5000× *g* for 10 min. The bacteria were washed with PBS and then fixed with 4% paraformaldehyde for 30 min. The bacteria were collected by centrifugation again, washed with PBS, resuspended in PBS solution with 0.3% Triton X-100, and incubated for 5 min. The bacteria were then washed twice with PBS and labelled using a One Step TUNEL Apoptosis Assay Kit (containing a mixture of terminal deoxynucleotidyl transferase and FITC-conjugated dUTP) (Beyotime, Shanghai, China). The FITC fluorescence was detected by a CytoFLEX flow cytometer.

#### 4.6.2. Flow Cytometric Analysis of Annexin V-FITC and Propidium Iodide (PI) Staining

*E. coli* was treated according to the description above, and then collected by centrifugation at 5000 g for 10 min. The bacteria were stained with Annexin V-FITC and propidium iodide according to the protocol of the Annexin V-FITC Apoptosis Detection Kit (Beyotime, Shanghai, China). The FL1 and FL2 signals were detected by a CytoFLEX flow cytometer.

### 4.7. Flow Cytometric Analysis of Annexin V-FITC and Propidium Iodide (PI) Staining

The experiment was carried out as reported by Vanhaecht et al [12]. In brief, a solution of a DNA simulant, 4-nitrophenyl phosphate disodium salt hexahydrate (NPP), was incubated with or without K_6_[P_2_Mo_18_O_62_] solution (20 mM) at 37 °C. Then, the NMR spectra of the mixed liquid were determined using an Avance Neo 600m full digital NMR spectrometer (Bruker, Billerica, MA, USA).

### 4.8. Statistical Analysis

The results were expressed as mean ± standard deviations (SD) and analyzed using a Student’s *t*-test or an analysis of variance (ANOVA) followed by Tukey’s test using Origin. *p*-values of less than 0.05 were considered as statistically significant.

## 5. Conclusions

In conclusion, the current study demonstrated that [P_2_Mo_18_O_62_] could effectively kill *E. coli* O157:H7 at millimolar levels. Moreover, the obtained results also showed that K_6_[P_2_Mo_18_O_62_] treatment triggered characteristic apoptosis-like bacterial death events such as *recA* overexpression, DNA fragmentation, and phosphatidylserine exposure. Taking these results together, polyoxometalate K_6_[P_2_Mo_18_O_62_] possessed a desirable antibacterial activity, and induction of bacterial apoptosis-like death might be involved its underlying bactericidal mechanisms.

## Data Availability

Data are available on request.
